# Alterations in the gut microbiota of patients with acquired immune deficiency syndrome

**DOI:** 10.1111/jcmm.13508

**Published:** 2018-02-07

**Authors:** Youlian Zhou, Zhitao Ou, Xiaoping Tang, Yongjian Zhou, Haoming Xu, Xianfei Wang, Kang Li, Jie He, Yanlei Du, Hong Wang, Ye Chen, Yuqiang Nie

**Affiliations:** ^1^ Department of Gastroenterology Guangzhou Digestive Disease Center Guangzhou First People's Hospital Guangzhou Medical University Guangzhou China; ^2^ Department of Internal Medicine Guangzhou No. 8 People's Hospital Guangzhou China; ^3^ Institute for Infectious Diseases Guangzhou No. 8 People's Hospital Guangzhou China; ^4^ Department of Gastroenterology Affiliated Hospital of North Sichuan Medical College Nanchong China; ^5^ State Key Laboratory of Organ Failure Research, Guangdong Provincial Key Laboratory of Gastroenterology Department of Gastroenterology Nanfang Hospital Southern Medical University Guangzhou China

**Keywords:** microbiota, dysbiosis, human immunodeficiency virus, acquired immune deficiency syndrome, transmission route, highly active antiretroviral therapy

## Abstract

Acquired immune deficiency syndrome (AIDS), caused by infection with human immunodeficiency virus (HIV), is associated with gastrointestinal disease, systemic immune activation and changes in the gut microbiota. Here, we aim to investigate the gut microbiota patterns of HIV‐infected individuals and HIV‐uninfected individuals in populations from South China. We enrolled 33 patients with HIV (14 participants treated with highly active antiretroviral therapy [HAART] for more than 3 months; the remaining 19 individuals had not received treatment) and 35 healthy controls (HC) for a cross‐sectional comparison of gut microbiota using stool samples. Gut microbial communities were profiled by sequencing the bacterial 16S rRNA genes. Dysbiosis was more common among patients with AIDS compared with healthy individuals. Dysbiosis was characterized by decreased α‐diversity, low mean counts of Bacteroidetes, *Faecalibacterium*,* Prevotella*,* Bacteroides vulgatus*,* Dialister* and *Roseburia inulnivorans*, and high mean counts of Proteobacteria, *Enterococcus, Streptococcus, Lactobacillus, Lachnociostridium, Ruminococcus gnavus* and *Streptococcus vestibularis*. Increased abundance of Bacilli was observed in homosexual patients. Proteobacteria were higher among heterosexual patients with HIV infections. Tenericutes were higher among patients with history of intravenous drug abuse. Restoration of gut microbiota diversity and a significant increase in abundance of Faecalibacterium, Blautia and Bacteroides were found in patients receiving HAART compared to those who did not receive. HIV infection‐associated dysbiosis is characterized by decreased levels of α‐diversity and Bacteroidetes, increased levels of Proteobacteria and the alterations of gut microbiota correlate with the route of HIV transmission. The imbalanced faecal microbiota of HIV infection is partially restored after therapy.

## Introduction

Infection with human immunodeficiency virus (HIV) is a chronic illness characterized by progressive loss of CD4^+^ T cells and chronic activation of the immune system. An estimated 35.3 million people worldwide are affected with HIV‐1, with more than 2 million new cases since 2012 (UNAIDS 2013). Increasing evidence suggests that the gut microbiome plays a crucial role in HIV transmission and pathogenesis 1, 2, 3. Understanding the interplay between the microbiome and HIV is of great value for developing effective strategies for the prevention and treatment of HIV.

The advancement of sequencing techniques and bioinformatics has allowed researchers to characterize microbial communities in health and disease states including HIV. Direct evidence of alterations in the composition of the gastrointestinal tract microbiome has been reported. Current data indicate increases in commensal bacteria that may be pathogenic (*e.g. Pseudomonas*) in the faeces of patients with HIV infection and decreases in primarily beneficial commensals such as *Lactobacilli* and *Bifidobacteria* 4. These changes suggest that HIV infection increases the risk for intestinal dysbiosis. Mutlu *et al*. 5 found a less diverse population of commensals in the mucosal microbiome inhabiting the right colon and terminal ileum of patients with HIV compared with control subjects. In addition, levels of bacterial products are increased in the circulation of patients with HIV 6; this can occur even at early stages of HIV infection, before peripheral CD4^+^ T‐cell depletion reaches levels that can lead to clinically apparent disease and opportunistic infections 4. Prebiotics have been administered to patients with HIV in an effort to improve the composition of the microbiome, resulting in reduced CD4^+^ T‐cell activation and improved natural killer (NK) cell activity 7.

However, most prior studies on gut microbiota in patients with HIV were performed in Western countries 2, 5, 8, 9, 10, 11, 12, 13, 14, 15, 16, 17, 18. The genetics, ethnic background, environment, dietary habits and lifestyles of such populations differ from those of comparable populations in China 19. Although little is known about alterations of gut microbiota in Chinese patients infected with HIV, Ling *et al*. researched the issue in Zhejiang province in eastern China. The authors observed that α‐diversity indices did not differ significantly between the healthy controls and patients infected with HIV‐1, while the proportion of Firmicutes/Bacteroidetes increased significantly in HIV‐1‐infected patients 20. Sun *et al*. 21. conducted research in Shanghai, also located in eastern China. The results showed that the microbiota of individuals infected with HIV had decreased α‐diversity, were enriched for Firmicutes and Proteobacteria at the phylum level and were depleted in families Ruminococcaceae and Lachnospiraceae and class Clostridia. While one study of Chinese populations from seven ethnic groups living in nine provinces found large ethnic and/or geographical differences in gut microbiota among individuals 22. To date, however, a detailed and comprehensive look at the gut microbiome in HIV‐infected individuals from South China has not yet been published. We therefore sought to identify bacterial microbiome patterns in HIV‐infected patients living in South China.

In this study, we examined the gut microbiota of healthy individuals and subjects from South China infected with HIV. We then investigated the relationship between the route of transmission of HIV in composition of the microbiome to identify a universal and specific biomarker for the development of acquired immune deficiency syndrome (AIDS). And we also enrolled HIV‐infected patients treated with highly active antiretroviral therapy (HAART) for ≥3 months to address any microbial differences among untreated HIV‐infected patients, HAART‐treated individuals and healthy controls. This investigation of microbial changes associated with HIV infection has the potential to aid in the prevention and development of therapeutic interventions that could improve many of the pathologic consequences of chronic HIV infection.

## Results

### Faecal bacterial diversity in patients with HIV infection

**Figure 1:**
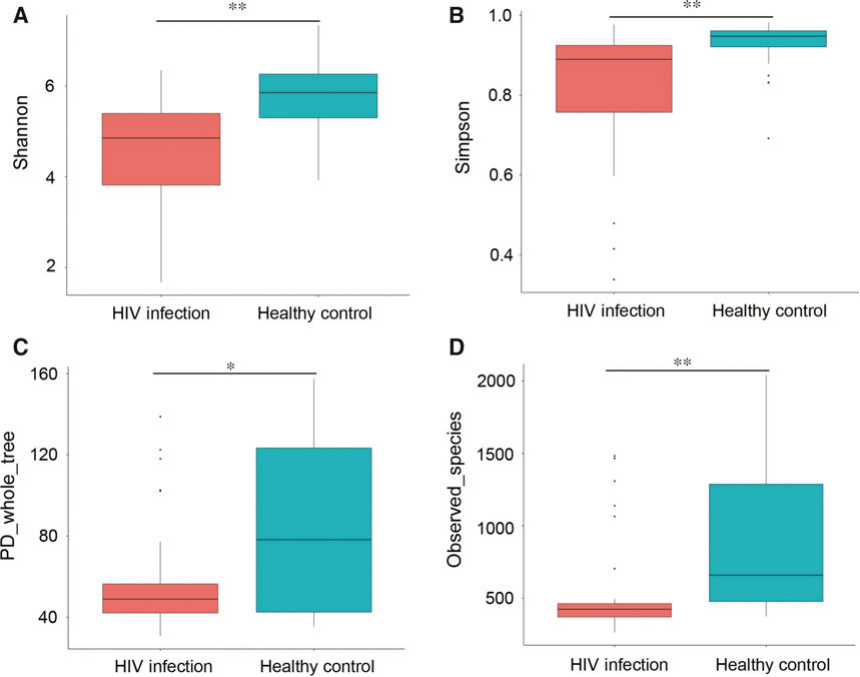
Αlpha diversity is decreased in HIV‐infected individuals. (**A**) Shannon index, (**B**) Simpson, (**C**) PD_whole tree, (**D**) observed species. *P < 0.05; **P < 0.01.

**Figure 2:**
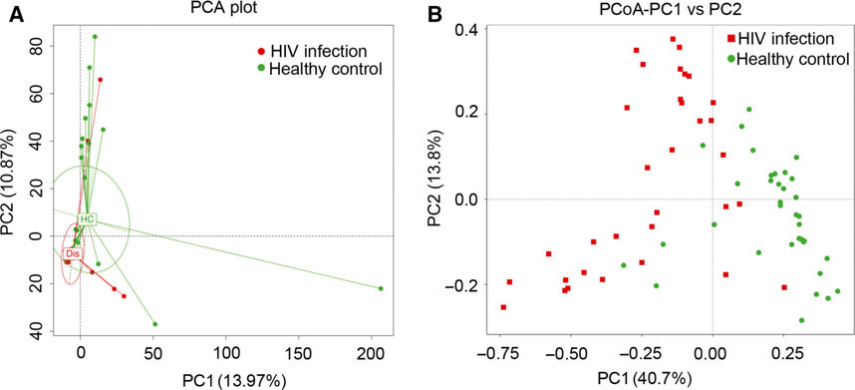
UniFrac‐based principal co‐ordinate analysis (PCoA) (**B**) and principal component analysis (PCA) (**A**) showing differences in the clustering of bacterial communities according to HIV infection status. Each dot represents a single faecal sample.

Alpha diversity comparison evaluates diversity, particularly taxa richness and evenness, within a habitat (or samples), while β‐diversity compares the similarity or difference in communities between habitats (or samples). We used four α‐diversity indices to compare the richness estimators (PD_whole tree and observed species) and diversity index (Simpson, Shannon index). The values presented represent the means with standard error for each group (Fig. 1). Our results showed that the α‐diversity of faecal microbiota was markedly reduced compared to healthy controls. To reveal the effect of HIV infection on analysis of microbiomes, we used a ß‐diversity comparison (PCA and PCoA), which reveals the similarity of microbiome community structures (Fig. 2). The results of PCA and PCoA showed that the microbiota of patients with HIV infection differed substantially from those of healthy individuals.

### Faecal bacterial composition in patients with HIV

**Figure 3:**
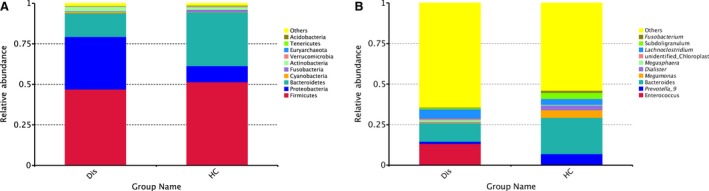
Relative abundance of faecal bacterial taxa (**A**) at the phylum level and (**B**) at the genus level. Abbreviations: Dis indicates HIV‐infection; HC indicates Healthy controls.

**Figure 4:**
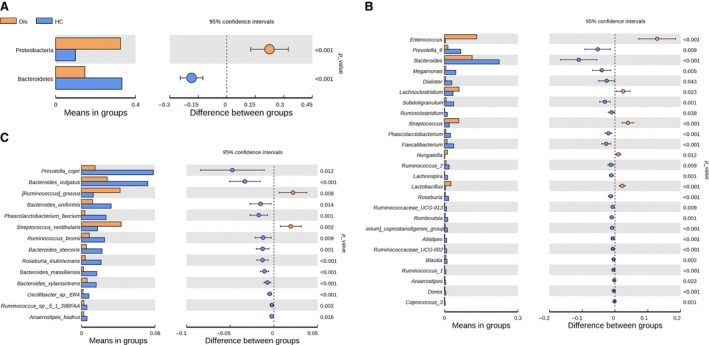
Faecal bacterial abundance at the phylum (**A**), genus (**B**) and species (**C**) levels.

We compared community structure among groups. Firmicutes, Proteobacteria, Bacteroidetes and Actinobacteria were the most predominant phyla, accounting for 97% of faecal samples, patients with HIV as well as controls (Figs 3A and 4A). The proportion of Proteobacteria increased in HIV‐infected patients compared with controls; the proportion of Bacteroidetes decreased in patients with HIV compared with controls. At the genus and species levels, *Enterococcus, Lachnoclostridium, Streptococcus, Lactobacillus, Ruminococcus* and *Streptococcus vestibularis* were most abundant in faecal microbiota in patients with HIV. In contrast, the proportionate representation of *Prevotella, Megamonas, Dialister, Ruminiclostridium, Faecalibacterium, Ruminococcus, Lachnospira, Roseburia, Blautia, Bacteroides vulgatus, Bacteroides uniformis, Phascolartobacterium faeclum, Ruminococcus bromii* and *Bacteroides stercoris* was markedly reduced in HIV‐infected patients (Figs 3B and 4B,C).

**Figure 5:**
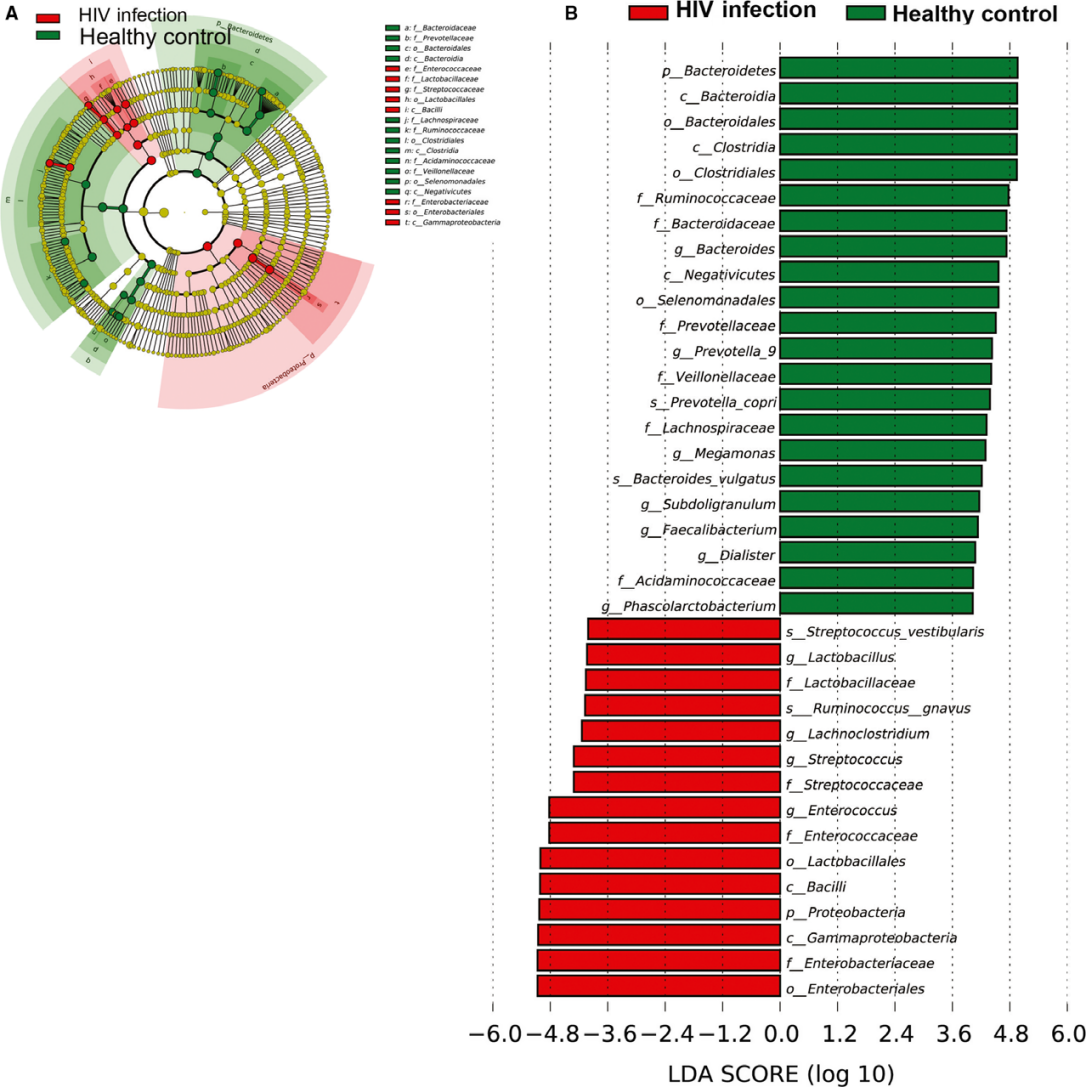
Taxonomic differences between the faecal microbiota of HIV‐infected patients and healthy controls by LEfSe and LDA. (**A**) LEfSe results for the bacterial communities, (**B**) cladogram using the LDA model results for the bacterial hierarchy.

To identify the specific bacterial taxa associated with HIV, we compared the composition of faecal microbiota in healthy controls and patients with HIV using linear discriminant analysis effect size (LEfSe). A cladogram that represents the structure of the faecal microbiota and the predominant bacteria in the healthy control and HIV‐positive patients is shown in Figure 5A. Changes in the composition of faecal microbiota in HIV‐1‐infected samples were also explored using the Mann–Whitney *U*‐test at different taxon levels. LEfSe analysis revealed 37 discriminative features (LDA score >3, Fig. 5B). Members of Bacteroidetes were enriched in the healthy‐control samples, whereas Firmicutes and Proteobacteria were enriched in the HIV‐positive patient samples. Firmicutes and Proteobacteria could therefore be used as biomarkers to identify patients with HIV infection.

### Effects of HIV transmission route on faecal microbiota

**Figure 6:**
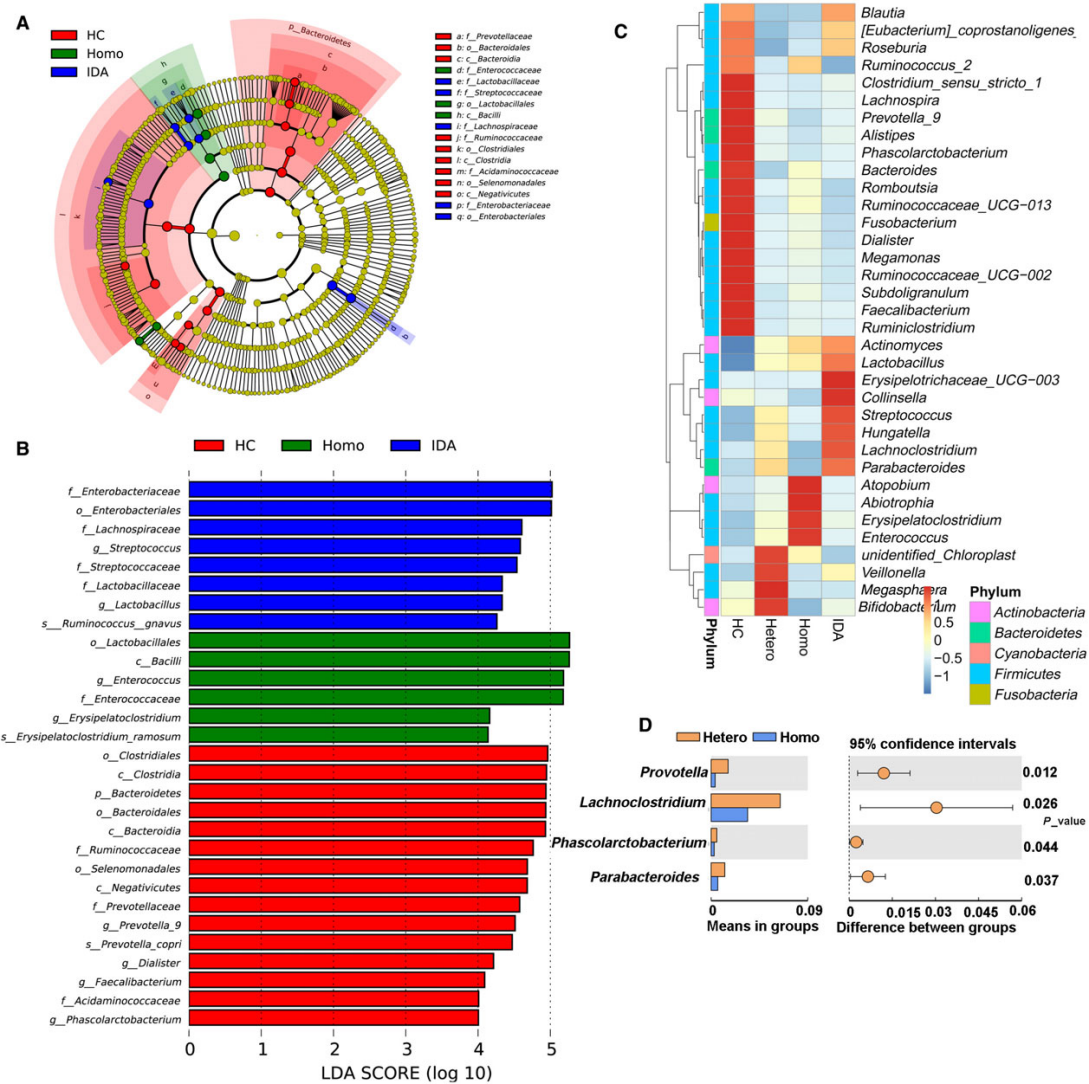
Effects of HIV transmission route on faecal microbiota in patients with HIV. (**A**) PCA plot, (**B**) relative abundance at the phylum level, (**C**) LEfSe results among HIV‐infected patients infected by different routes of transmission and (**D**) relative abundance bacteria with significance between heterosexual (Hetero) and homosexual (Homo) subjects. Abbreviations: HC, Healthy controls; Homo, Homosexual; Hetero, heterosexual; IDA, intravenous drug abuse.

HIV patients were divided into three groups according to transmission route: heterosexual, homosexual or intravenous drug abuse (IDA). We then analysed alterations of the gut microbiota among these three groups. Results (Fig. 6) showed that several microbial signatures in the faecal microbiota differed among homosexual HIV‐infected patients, intravenous drug abusers with HIV and healthy individuals. Bacilli, Lactobacillales and Enterococcaceae were significantly more abundant in the faeces of HIV‐infected patients infected by homosexual transmission, whereas Enterobacteriales, Enterobacteriaceae, Lachnospiraceae, Streptococcaceae and Lactobacillaceae were significantly more abundant in faecal samples from HIV‐infected patients infected through IDA. *Provotella*,* Lachnoclostridium*,* Phascolarctobacterium* and *Parabacteroides* were markedly more abundant in heterosexual as compared with homosexual patients (Fig. 6D).

### Partial restoration of gut microbiota in HIV‐infected patients after HAART

**Figure 7:**
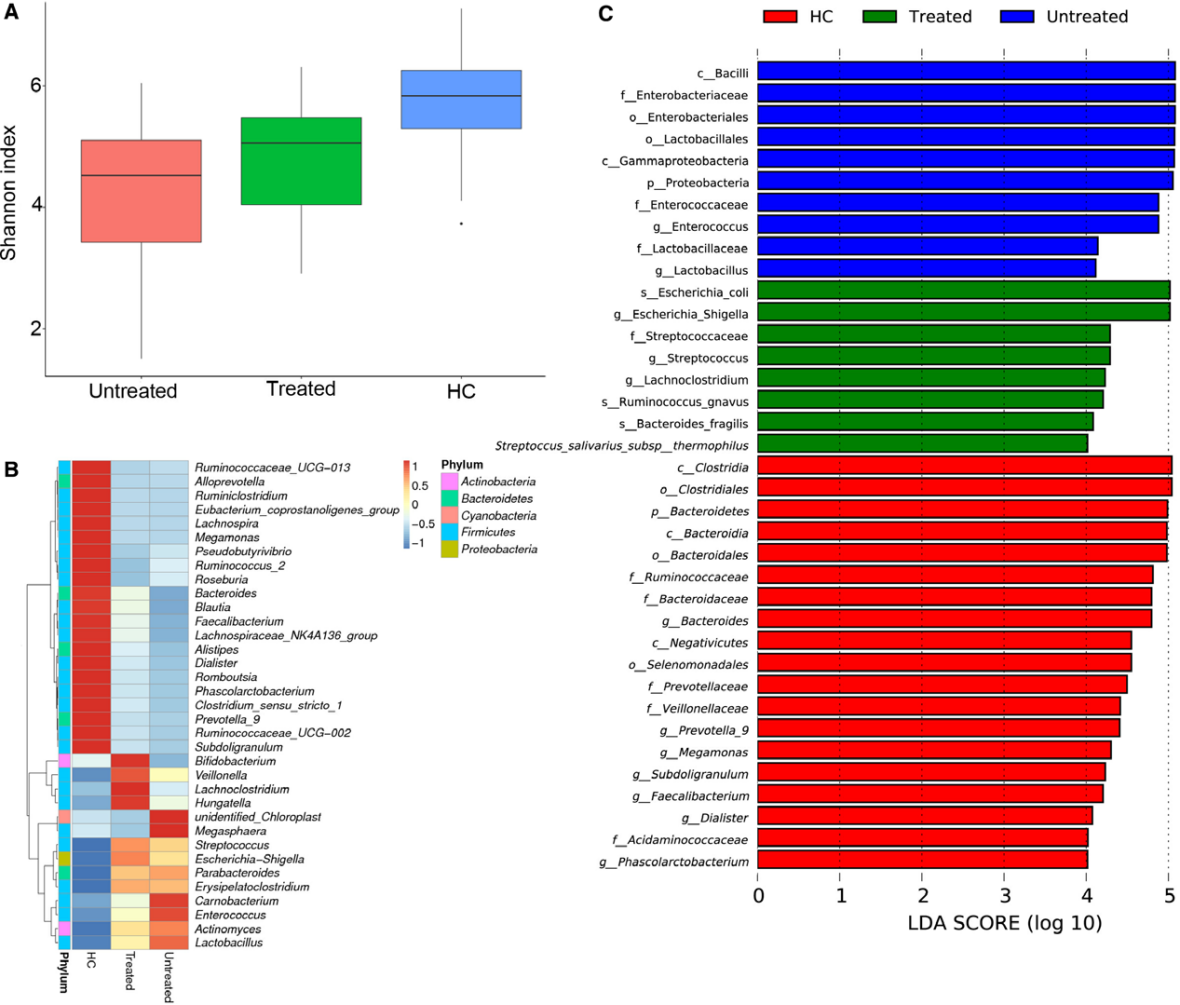
Effects of HAART on gut microbiota in patients with HIV. (**A**) Bacterial diversity by Shannon index. (**B**) Heatmap showing abundance distribution of the OTUs identified as key variables among HC, HAART‐treated and untreated HIV individuals. (**C**) LDA results for the bacterial hierarchy.

We also enrolled 14 HIV‐infected patients treated with HAART for ≥3 months to identify any microbial differences among untreated HIV‐infected patients, HAART‐treated individuals and healthy controls. The results showed that microbial diversity (as measured with the Shannon index) was increased after HAART; these effects were most apparent as increased levels of Bacteroides, Blautia and Faecalibacterium (Fig. 7). Levels of bacterial taxa such as *Rminccoccaceae, Ruminiclostridium, Eubacterium_coprostanoligenes, Lachnospira* and *Roseburia* showed no change. Thus, despite the efficacy of HAART, the faecal microbiota of HIV‐1‐infected patients was not completely restored after therapy.

## Discussion

The present study is the first to compare gut microbiota in HIV‐infected patients with those of age‐ and gender‐matched healthy individuals in a population from South China using sequencing technologies. Our data show that the intestinal bacterial communities in HIV‐infected patients are less diverse and distinct from those of non‐HIV controls. In patients with HIV, the gut microbiota typically included higher proportions of potentially pathogenic microbes such as Proteobacteria, *Enterococcus, Lachnoclostridium, Streptococcus, Lactobacillus and Ruminococcus*. The gut microbiota of patients with HIV, compared with those of controls, contained lower levels of Bacteroidetes, *Prevotella, Megamonas, Dialister, Ruminiclostridium, Faecalibacterium, Ruminococcus, Lachnospira, Roseburia, Blautia, Bacteroides vulgatus, Bacteroides uniformis, Phascolartobacterium faeclum, Ruminococcus bromii* and *Bacteroides stercoris*.

Changes to the gut microbiota during infection with HIV are influenced by factors such as study population, race, geography, dietary habits, lifestyle, sex, age, sample type and treatment. The complexity of the issue has led to methodological discrepancies between studies that make it difficult to compare results. Dillon *et al*. 2. observed an increased abundance of the phylum Proteobacteria in HIV‐infected individuals was only noted with mucosal samples, suggesting that luminal samples may miss potentially pathogenic alterations. However, in the present study, we also observed an increased abundance of the phylum Proteobacteria in HIV‐infected patients with faecal samples. Samples were collected from the mid‐stream stool in the present study, which is less convenient than swabbing, but may retain the signal of microbial changes better, because of the biogeographic heterogeneity in the stool.

Several studies have found that α‐diversity was significantly decreased in untreated HIV‐infected patients 9, 14. While Ling *et al*. 20 showed no difference in bacterial alpha diversity of gut microbiota between HIV‐infected patients and healthy controls, another study found a clear decrease in overall bacterial diversity after effective HAART 5, 8. In this study, HIV‐infected individuals had lower levels of *Prevotella,* a genus of Gram‐negative anaerobic bacteria previously grouped in the genus *Bacteroides* and *Bacteroides*. These findings are inconsistent with previous studies, although previous studies largely focused on populations from Western countries 2, 5, 9, 13. This variation in composition of the gut microbiota might reflect differences in diet or host genetic background between Chinese and Western populations. The Western diet is high in fat and calories, while Chinese diets typically include low levels of fat, sugar and meat, which may have significant effects on the gut microbiota. Kashyap *et al*. 26 have suggested that different dietary patterns are strongly associated with gut microbiota enterotypes. Lozupone *et al*. 8. have shown that diets high in fat and protein and low in carbohydrates and fibre diets are correlated with the loss of beneficial bacteria in HIV‐infected patients.

This study also provided a comparison of gut microbiota among HIV‐infected patients infected by different transmission routes to identify key biomarkers that might be associated with HIV infection. In the largest study to date examining the gut microbiota of HIV‐infected individuals, Noguera‐Julian *et al*. 14. found that a high Prevotella/low Bacteroides enterotype in stool specimens was highly associated with men who have sex with men (MSM) behaviour, regardless of HIV‐1 infection status. This may explain the perceived association between this enterotype and HIV infection status in prior studies that did not control for sexual behaviour. In the present study, several microbial signatures in the faecal microbiota differed among homosexual HIV‐infected patients, intravenous drug abusers with AIDS and healthy individuals. Although our results were not completely consistent with previous studies, this is the first attempt, to the best of our knowledge, to compare microbiota communities among Chinese individuals infected with HIV through different routes of transmission. This study shows that gut microbiota may be used to help prevent and treat HIV.

A few limitations to the present study should be acknowledged. In an observational study such as this, it is not possible to untangle the causal relationship between gut microbiota and HIV. As samples were collected from patients who had already been diagnosed with HIV, changes in the gut microbiota may have been a cause or a consequence of HIV. HIV infection disrupts host–microbe interactions such as the mucosal immune response, which may have confounded our results. Secondly, the sample size for this study, especially the subgroups of HIV‐infected individuals stratified by transmission route, were rather small. In addition, because of the lack of data on follow‐up, we could not evaluate the gut microbial changes that may be associated with effective antiretroviral therapy.

In summary, our study reveals alterations in the gut microbiome and dysbiosis in HIV‐infected patients from South China. These findings may aid the establishment of principles guiding HIV management. Our results confirm that the gut microbiome contains promising biomarkers for the non‐invasive evaluation of routes of HIV transmission. The identification of microbiota associated with specific routes of HIV transmission represents one step towards establishing a set of microbiota‐based biomarkers for the assessment of HIV. Prospective studies are needed to evaluate the long‐term consequences of alterations of the microbiota that accompany HIV infection, the host's immune response and HIV treatment.

## Conflicts of interest

None declared.

## Author contributions

ZYL involved in design of the study, recruitment of patients, statistical analysis and interpretation of the data, and drafting of the article; OZT involved in recruitment of patients, statistical analysis and interpretation of the data; TXP revised the article; XHM involved in sample collection and DNA extraction; WXF, LK, HJ, DYL, ZYJ and WH involved in interpretation of the data and revision of the article; CY involved in interpretation of the data and revision of the article; NYQ involved in concept and design of the study, interpretation of the data and revision of the article.
